# *Lactobacillus fermentum* ATCC 23271 Displays *In vitro* Inhibitory Activities against *Candida* spp.

**DOI:** 10.3389/fmicb.2016.01722

**Published:** 2016-10-27

**Authors:** Monique S. do Carmo, Francisca M. F. Noronha, Mariana O. Arruda, Ênnio P. da Silva Costa, Maria R. Q. Bomfim, Andrea S. Monteiro, Thiago A. F. Ferro, Elizabeth S. Fernandes, Jorge A. Girón, Valério Monteiro-Neto

**Affiliations:** ^1^Centro de Ciências Biológicas e da Saúde, Universidade Federal do MaranhãoSão Luís, Brazil; ^2^Centro de Ciências da Saúde, Universidade CEUMASão Luís, Brazil; ^3^Vascular Biology and Inflammation Section, Cardiovascular Division, King’s College LondonLondon, UK; ^4^Centro de Detección Biomolecular, Benemérita Universidad Autónoma de PueblaPuebla, Mexico

**Keywords:** *Lactobacillus fermentum*, probiotic, genital infections, STD, *Candida*

## Abstract

Lactobacilli are involved in the microbial homeostasis in the female genital tract. Due to the high prevalence of many bacterial diseases of the female genital tract and the resistance of microorganisms to various antimicrobial agents, alternative means to control these infections are necessary. Thus, this study aimed to evaluate the probiotic properties of well-characterized *Lactobacillus* species, including *L. acidophilus* (ATCC 4356), *L. brevis* (ATCC 367), *L. delbrueckii* ssp. *delbrueckii* (ATCC 9645), *L. fermentum* (ATCC 23271), *L. paracasei* (ATCC 335), *L. plantarum* (ATCC 8014), and *L. rhamnosus* (ATCC 9595), against *Candida albicans* (ATCC 18804), *Neisseria gonorrhoeae* (ATCC 9826), and *Streptococcus agalactiae* (ATCC 13813). The probiotic potential was investigated by using the following criteria: (i) adhesion to host epithelial cells and mucus, (ii) biofilm formation, (iii) co-aggregation with bacterial pathogens, (iv) inhibition of pathogen adhesion to mucus and HeLa cells, and (v) antimicrobial activity. Tested lactobacilli adhered to mucin, co-aggregated with all genital microorganisms, and displayed antimicrobial activity. With the exception of *L. acidophilus* and *L. paracasei*, they adhered to HeLa cells. However, only *L. fermentum* produced a moderate biofilm and a higher level of co-aggregation and mucin binding. The displacement assay demonstrated that all *Lactobacillus* strains inhibit *C. albicans* binding to mucin (*p* < 0.001), likely due to the production of substances with antimicrobial activity. Clinical isolates belonging to the most common *Candida* species associated to vaginal candidiasis were inhibited by *L. fermentum*. Collectively, our data suggest that *L. fermentum* ATCC 23271 is a potential probiotic candidate, particularly to complement candidiasis treatment, since presented with the best probiotic profile in comparison with the other tested lactobacilli strains.

## Introduction

The microbiota of the female genital tract is composed of diverse aerobic, anaerobic, and microaerophilic microorganisms (Ravel et al., 2011). The genus *Lactobacillus* is considered a biological marker of the normal vaginal microbiota (Lidbeck and Nord, 1993; Witkin et al., 2007). The majority of reproductive-aged women carry 10^7^–10^9^ *Lactobacillus*/g of vaginal secretion (Ferris et al., 2007; Zhou et al., 2007). Lactobacilli display several defense mechanisms against pathogens, such as the production of antimicrobial components and adhesion to the mucosa to create a barrier that inhibits pathogen colonization by competing for binding sites on epithelial cells (Walencka et al., 2008; Medellin-Pena and Griffiths, 2009; Nami et al., 2014; Gorska et al., 2016).

Several factors or conditions can alter the balance of the genital tract microbiota, including inadequate or prolonged antibiotic therapy, tight or synthetic-fiber clothing and an acidic diet. Such conditions facilitate the excessive growth of aerobic and anaerobic microorganisms in the genital tract, leading to the development of two clinical syndromes, vaginitis and vaginosis, which are very common among females (Ravel et al., 2011). Vaginitis is characterized by the presence of inflammation and discharge, which are triggered by the proliferation of some pathogens, especially *Candida albicans*, amongst others (Donders et al., 2002; Lobos et al., 2013; Wang et al., 2016).

In addition to these microorganisms, several agents of sexually transmitted diseases (STDs) represent a serious public health burden due to their high morbidity rates. For example, infections due to *Neisseria gonorrhoeae* are among the most prevalent STDs. The World Health Organization reports 106 million gonorrhea new cases annually. Gonococcal infections that are not adequately treated may cause permanent damage to the female reproductive tract, resulting in ectopic pregnancy or even infertility (WHO, 2012).

Other microorganisms, such as *Streptococcus agalactiae*, colonize the female genital tract as commensals of the vagina, but can be transmitted to newborns during delivery (Hansen et al., 2004), leading to neonatal infection and meningitis (Puopolo et al., 2005). Although antibiotic prophylaxis has decreased the incidence of these infections, case reports of disease caused by *S. agalactiae* continue to occur in infants with significant morbidity and mortality (Puopolo et al., 2005). Therefore, new alternative approaches are needed, since the current methods to prevent genital infections are not always effective against those caused by primary pathogens or commensal microorganisms transmitted during birth.

Bacterial adhesion to the mucosa and epithelial cell surface is a key event in the colonization of the host and disease progression. Understanding the adhesion strategies used by pathogens in order to colonize the host is crucial for the development of preventive or therapeutic interventions against infectious diseases. One approach is the use of non-pathogenic bacteria that are natural components of the microbiota and that can potentially inhibit the adhesion and establishment of pathogenic bacteria (Spurbeck and Arvidson, 2010).

Members of the *Lactobacillus* genus have been proposed as probiotics as they can protect against infectious disease agents (Li et al., 2012; Ren et al., 2012; Zabihollahi et al., 2012; Alexandre et al., 2014). From those, some (*L. crispatus* and *L. acidophilus*) commonly colonize the female genital tract and have been studied as probiotics (Mastromarino et al., 2002; Antonio et al., 2009; Zhang et al., 2012; Kaewnopparat et al., 2013; Nami et al., 2014; Verdenelli et al., 2014). However, it is not known whether *Lactobacillus* species that are less frequent in the vagina also display probiotic activities. Here, we evaluated the probiotic potential of several reference species of the *Lactobacillus* genus not usually found in the vaginal microbiome against three important microorganisms associated with genital infections, neonatal sepsis, and meningitis.

## Materials and Methods

### Bacterial Strains and Growth Conditions

The following *Lactobacillus* reference strains were used: *L. brevis* (ATCC 367), *L. delbrueckii* ssp. *delbrueckii* (ATCC 9645), *L. fermentum* (ATCC 23271), *L. paracasei* (ATCC 335), *L. plantarum* (ATCC 8014), and *L. rhamnosus* (ATCC 9595). For comparison, a reference strain of *Lactobacillus acidophilus* (ATCC 4356) was included in the study as a representative species of the female genital tract. All strains were routinely grown in Man, Rogosa, and Sharpe (MRS) broth or agar (Difco, USA) and incubated in an anaerobic atmosphere (Probac, Brazil) at 37°C for 24 h. The pathogens evaluated were: (i) *C. albicans* (ATCC 18804), which was grown on Sabouraud dextrose agar (Difco, USA) at 37°C for 24 h; (ii) *N. gonorrhoeae* (ATCC 9826), which was cultured on gonococci (GC) agar (Difco, USA) supplemented with VX supplement (LaborClin, Brazil) and hemoglobin (Difco, USA) at 37°C for 48 h under a 5% CO_2_ atmosphere; and (iii) *S. agalactiae* (ATCC 13813), which was cultivated on Columbia agar (Difco, USA) supplemented with 5% sheep blood, under 5% CO_2_ at 37°C for 24 h. All strains were obtained from the Culture Collection Laboratory of the Fundação Instituto Oswaldo Cruz (Rio de Janeiro, Brazil) and stored in Brain Heart Infusion (BHI) broth (Difco, USA) with 20% glycerol (Amresco, Australia) at -86°C. Clinical isolates of *Candida* spp. were kindly donated by Dr. Cristina A. Monteiro from the Universidade CEUMA.

### HeLa Cell Culture

Human cervical epithelial (HeLa) cells were cultured in Dulbecco’s Modified Eagle’s Medium (DMEM) containing GlutaMAX (Gibco^TM^, USA) and supplemented with 1× antibiotic-antimycotic solution (Gibco, USA) and 10% fetal bovine serum (Gibco, USA) at 37°C under a 5% CO_2_ atmosphere.

### Adhesion Assays to Eukaryotic Cells

Prior to the adhesion assays, *Lactobacillus* strains were cultured in MRS broth under the conditions described above. After 24 h, the bacterial cultures were centrifuged at 6,000 × *g* and washed three times with phosphate-buffered saline (PBS, pH 7.4, Sigma, USA) in order to remove organic acids produced during growth. The pellets were resuspended in 300 μl of DMEM. The adhesion assay was performed as previously described (Kaewnopparat et al., 2013), with minor modifications. Briefly, HeLa cell monolayers were seeded into 24-well plates with or without glass coverslips and allowed to grow to 80–90% confluence. Fifty microliters (~2.3 × 10^7^) of the bacterial suspensions were added to each well, and the plates were incubated at 37°C under 5% CO_2_ for 3 h. Then, each well was washed three times with PBS for removal of non-adherent bacteria. Immediately after, the cells were incubated with 0.1% Triton X-100 (Sigma, USA) for 5 min, and 10-fold serial dilutions were spread onto agar plates for quantification of the total number of bacteria adhered to the cells (in colony forming unit; CFU). To visualize the bacterial adherence to HeLa cells, each glass coverslip was fixed with methanol (P.A., Amresco, Australia) and stained with 0.2% May-Grunwald and 0.6% Giemsa (Amresco, Australia). The coverslips were then mounted onto glass slides and visualized by light microscopy under oil-immersion objective 100× (Leica ICC50 HD microscope). HeLa cells incubated in the absence of bacteria were used as negative controls.

### Biofilm Formation by *Lactobacillus* spp.

Biofilm formation on a polystyrene surface was quantified in 96-well plates as described by Stepanovic et al. (2000). Briefly, *Lactobacillus* spp. were cultured for 24 h and then adjusted to an optical density of 0.7 at 540 nm. Then, 200 μl of the bacterial suspensions were added into the wells. Wells containing MRS broth only were used as negative controls. The plates were incubated at 37°C for 24 h, washed three times with 300 μl of PBS (pH 7.4) and air-dried. Each well was then fixed with 200 μl of methanol (P.A., Amresco, Australia) and the plate was incubated at room temperature for 15 min. Following, the methanol was discarded, and the microplate was air-dried. Two hundred microliters of 2% crystal violet (Amresco, Australia) was added to each well and incubated for 15 min. The plate was then washed three times with distilled water and air-dried. To extract the dye impregnated in the biofilm, 160 μl of 33% glacial acetic acid (Amresco, Australia) were added per well. The absorbance was immediately measured at 540 nm using an ELISA plate reader (Bio-Tek Instrument Co., WA, USA) and taken as indicative of biofilm formation. Based on the optical densities of the isolates (OD) and the negative controls (OD_C_), the biofilm formation by *Lactobacillus* was classified as follows: 1, non-adherent: OD ≤ OD_C_; 2, weakly adherent: OD_C_ < OD ≤ (2 × OD_C_); 3, moderately adherent: (2 × OD_C_) < OD ≤ (4 × OD_C_); 4, strongly adherent: (4 × OD_C_) < OD.

### Hydrophobicity of *Lactobacillus* spp. Cell Surface

The hydrophobicity of *Lactobacillus* cell surface was evaluated using the salt aggregation assay (Andreu et al., 1995). For this, bacteria were resuspended in 0.02 M sodium phosphate (pH 6.8). Bacterial suspensions (~1.2 × 10^9^ CFU/ml) were mixed (1:1, v/v) with different concentrations of ammonium sulfate (0.5–4.0 M; Amresco, Australia) on a glass slide. The lowest final concentration of ammonium sulfate able to promote aggregation of *Lactobacillus* was defined as the salt aggregation value. Hydrophobicity was classified as high, intermediate, or hydrophilic at salt aggregation values of <0.9, 0.9–1.5, and >1.5 M, respectively. For comparison, a solution of 0.02 M sodium phosphate (pH 6.8) without bacteria was used as negative control.

### Binding of *Lactobacillus* spp. and Genital Pathogens to Mucin

The ability of the pathogen and *Lactobacillus* strains to adhere to mucin was evaluated as previously described (Tallon et al., 2007), with minor modifications. Initially, porcine gastric mucin-type III (Sigma, Saint-Quentin-Fallavier, France) was solubilized in PBS (Sigma, USA; pH 7.4) at a final concentration of 10 mg/ml. Five hundred microliters of mucin solution were added into a 24-well plate and allowed to bind to the wells for 16 h at 4°C. The mucin solution was removed; the wells were washed three times with 1 ml of PBS, saturated with 2% bovine serum albumin for 4 h at 4°C, and washed four times with PBS. Bacterial cultures were centrifuged at 6,000 × *g* for 5 min, washed three times with PBS, and resuspended in the same buffer to an optical density of 0.1 at 600 nm (~4.5 × 10^7^ CFU/ml for lactobacilli and ~1.5 × 10^8^ CFU/ml for pathogen strains). Next, 500 μl of each bacterial suspension were added to the wells, and the plate was incubated at 37°C for 1 h. After incubation, the wells were washed three times with 1 ml of PBS to remove unbound bacteria. In order to quantify the number of bound bacteria, each well was treated with 1 ml of 0.5% Triton X-100, and the plates were incubated for 30 min at room temperature under agitation. Aliquots (100 μl) were taken from each well, and 10-fold serial dilutions were prepared and spread onto agar plates for quantification of the total number of bacteria adhered to mucin (in CFU). For all experiments, the purity of the bacterial colonies was checked both macroscopically (by the growth aspects) and microscopically (by the Gram stain). Mucin-containing wells without bacteria were used as negative controls.

### Exclusion and Displacement Inhibition Assays

After determining which organisms are able to adhere to mucin, we performed both exclusion and displacement inhibition assays. For this, *Lactobacillus* spp. (~4.5 × 10^7^ CFU/ml) were inoculated either 1 h prior (exclusion assay) or after (displacement assay) the tested pathogens (~1.5 × 10^8^ CFU/ml) (Tallon et al., 2007). Quantification of *Lactobacillus* and genital pathogens was carried out as described above (see Binding of *Lactobacillus* spp. and Genital Pathogens to Mucin). Mucin-containing wells without bacteria were used as negative controls.

### Co-aggregation of *Lactobacillus* spp. with Genital Pathogens

This assay was performed to determine whether the lactobacilli are able to co-aggregate with the tested genital pathogens. The lactobacilli and pathogen suspensions (in PBS) were adjusted to achieve turbidity equivalent to that of McFarland’s N^o^. 4 standard (~1.2 × 10^9^ CFU/ml). Five hundred microliters of each *Lactobacillus* were added per well and mixed with 500 μl of each pathogen suspension into 24-well plates. The plates were then incubated at 37°C for 4 h under constant agitation (100 rpm) in an orbital shaker. The microbial suspension of each well was evaluated under an inverted light microscope and a score was attributed as described by Reid et al. (1990): 0 = no aggregation, 1 = small aggregates comprising small visible clusters of bacteria, 2 = aggregates comprising larger numbers of bacteria, settling to the center of the well, 3 = macroscopically visible clumps comprising larger groups of bacteria which settle to the center of the well, 4 = maximum score allocated to describe a large, macroscopically visible clump in the center of the well. The aggregates were visualized using an inverted light microscope with a 16× magnification lens. Accordingly, results were expressed as aggregation score.

### Antimicrobial Activity of *Lactobacillus* spp. against Genital Pathogens

The antimicrobial actions of *Lactobacillus* spp. against genital pathogens were assessed as previously described by Mastromarino et al. (2002) with minor modifications. For this, 10 μl (~10^7^ CFU/ml) of *Lactobacillus* spp. suspensions were spotted onto MRS agar, and bacteria were allowed to grow for 24 h at 37°C, under anaerobiosis. In a typical assay, three to four distinct suspensions of *Lactobacillus* strains were spotted onto a 90 mm-diameter plate. After incubation, 10 ml of the specific culture medium for each pathogen was cooled to 50°C and poured onto the plate. Pathogen suspensions were adjusted to achieve turbidity equivalent to that of McFarland’s N^o^. 0.5 standard (~1.5 × 10^8^ CFU/ml, for bacterial pathogens and 1–5 × 10^6^ CFU/ml, for *C. albicans*). Pathogens were then spread separately onto agar plates and incubated for 24 or 48 h at 37°C under aerobic or 5% CO_2_ culture conditions, depending on the microorganism. Antimicrobial activities were characterized by the diameter (in millimeter) of the zone of growth inhibition directly above the *Lactobacillus* growth area.

### Statistical Analysis

GraphPad Prism (version 5.0; GraphPad Software, Inc. San Diego, CA, USA) software was employed for statistical analysis. For the mucin adhesion and inhibition assays, the Shapiro–Wilk normal test and analysis of variance (ANOVA) test were employed. The Dunnett’s test was employed to compare different groups of microorganisms with respect to the control. Statistical significance was established at *p* < 0.05. All assays were performed in triplicate on three independent days.

## Results

### Adhesion Ability of *Lactobacillus* spp. to HeLa Cells

**FIGURE 1:**
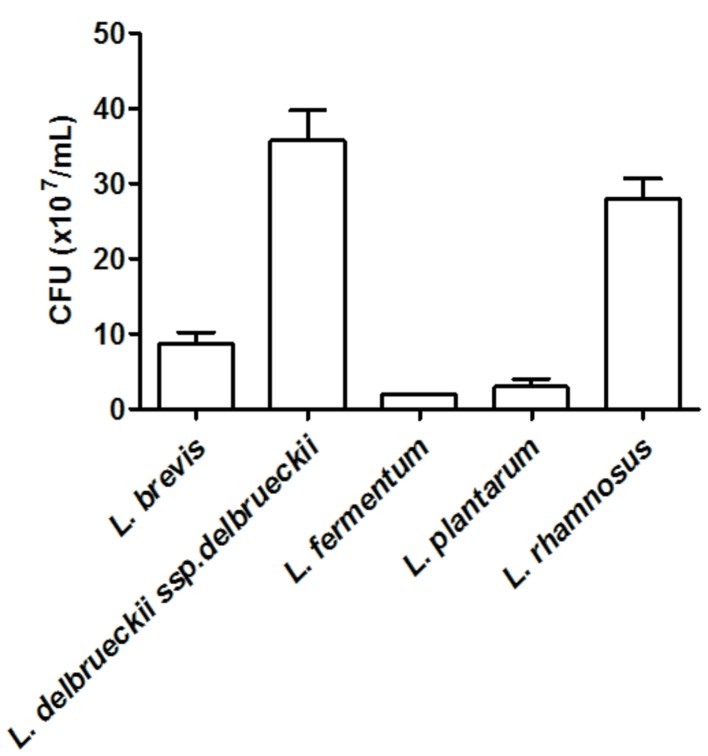
**Quantification of the adhesion of *Lactobacillus* (~2.3 × 10^6^ CFU) strains to HeLa cells.** The values are expressed in CFU/ml and represent the means of the values obtained. The assay was performed in triplicate.

We investigated the ability of *Lactobacillus* spp. to adhere to human epithelial (HeLa) cells. Amongst the tested *Lactobacillus*, *L. brevis* ATCC 367, *L. delbrueckii* ssp. *delbrueckii* ATCC 9645, *L. fermentum* ATCC 23271, *L. plantarum* ATCC 8014, and *L. rhamnosus* ATCC 9595 displayed the highest levels of adhesion to epithelial cells, as denoted by the number of bacteria adhered to the cells, which ranged from 10^7^ to 10^8^ CFU/ml (**Figure 1**). On the other hand, *L. acidophilus* ATCC 4356 and *L. paracasei* ATCC 335 presented the lowest capacity to adhere to HeLa cells, with a recovery of less than 10^3^ CFU/ml from the cell surface.

### Hydrophobicity of *Lactobacillus* spp. Cell Surface and Biofilm Formation

Overall, none of the tested lactobacilli were great biofilm producers with only *L. fermentum* presenting a moderate ability to form biofilm on a plastic surface (**Table 1**). *L. acidophilus* was unable to form biofilm under the used experimental settings. Also, with the exception of *L. acidophilus*, all strains exhibited high cell surface hydrophobicity (**Table 1**).

**Table 1:** Biofilm formation and hydrophobicity of *Lactobacillus* spp. cell surface.

*Lactobacillus* species	Biofilm^∗^	Hydrophobicity^∗∗^
*L. acidophilus*	Non-adherent	Hydrophilic
*L. brevis*	Weakly adherent	High
*L. delbrueckii* ssp. *delbrueckii*	Weakly adherent	High
*L. fermentum*	Moderately adherent	High
*L. paracasei*	Weakly adherent	High
*L. plantarum*	Weakly adherent	High
*L. rhamnosus*	Weakly adherent	High

*^∗^After measuring the optical densities of the isolates (OD) and the negative control (OD_C_), the biofilms were classified as: 1, non-adherent: OD ≤ OD_C_; 2, weakly adherent: OD_C_ < OD ≤ (2 × OD_C_); 3, moderately adherent: (2 × OD_C_) < OD ≤ (4 × OD_C_); 4, strongly adherent: (4 × OD_C_) < OD. The assays were performed in triplicate. ^∗∗^Lactobacillus hydrophobicity was classified as high, intermediate, or hydrophilic at salt aggregation values of <0.9, 0.9–1.5, and >1.5 M, respectively.*

### Binding of *Lactobacillus* spp. and Genital Pathogens to Mucin

**FIGURE 2:**
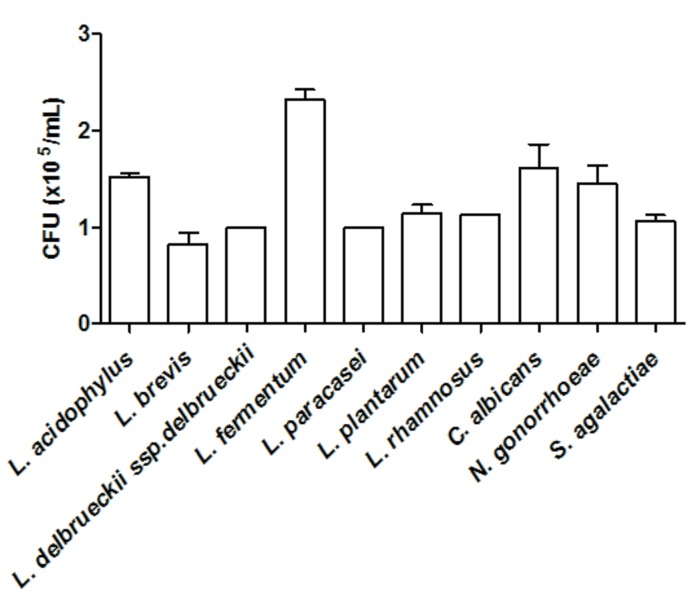
**Quantification of mucin adhesion by *Lactobacillus* spp. (~4.5 × 10^7^ CFU/ml) and genital pathogens (~1.5 × 10^8^ CFU/ml).** The values are expressed in CFU/ml and represent the means of the values obtained. The assay was performed in triplicate.

**FIGURE 3:**
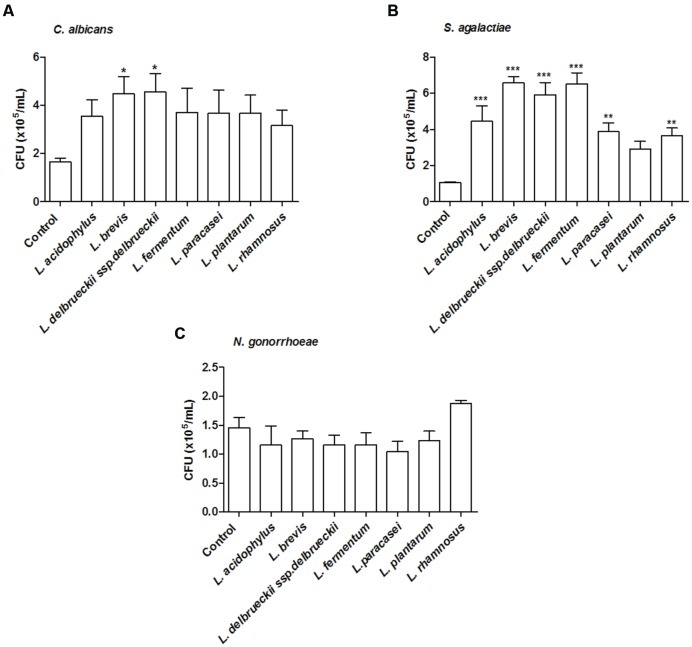
**Inhibition of mucin adhesion by exclusion.** Effect of different lactobacilli on **(A)** *C. albicans*, **(B)** *S. agalactiae*, and **(C)** *N. gonorrhoeae* binding to mucin. The values are expressed in CFU/ml and represent the means of the values obtained. For all experiments, the purity of the colonies was checked both macroscopically (by the growth aspects) and microscopically (by the Gram stain). The assay was performed in triplicate. ^∗^*p* < 0.05; ^∗∗^*p* < 0.01; ^∗∗∗^*p* < 0.001.

**FIGURE 4:**
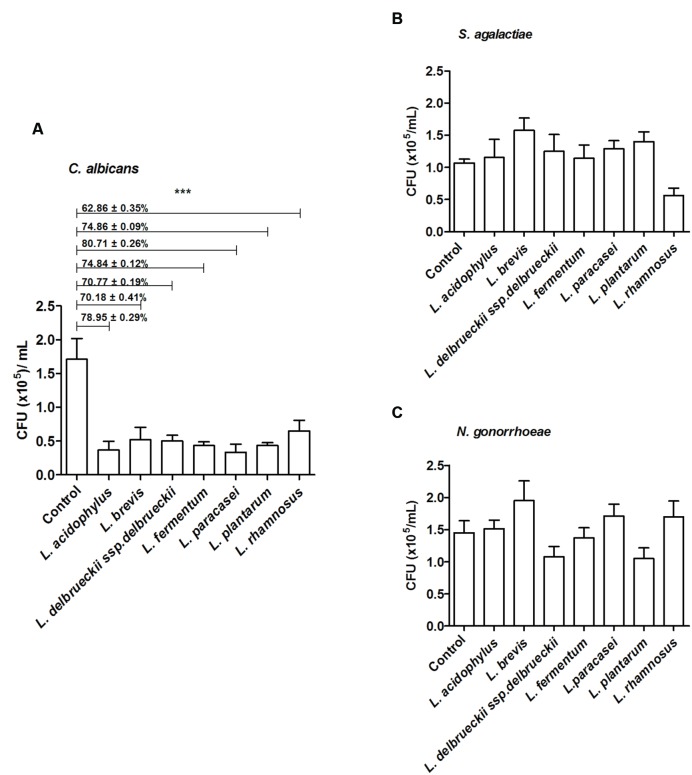
**Inhibition of mucin adhesion by displacement.** Effect of different lactobacilli on **(A)** *C. albicans*, **(B)** *S. agalactiae*, and **(C)** *N. gonorrhoeae* binding to mucin. The values are expressed in CFU/ml and represent the means of the values obtained. For all experiments, the purity of the colonies was checked both macroscopically (by the growth aspects) and microscopically (by the Gram stain). The assay was performed in triplicate. ^∗∗∗^*p* < 0.001.

All tested microorganisms presented similar ability to bind to mucin, as the number of cells recovered at the end of the assay was greater than 10^4^ CFU/ml for both the lactobacilli and the genital pathogens (**Figure 2**). Interestingly, *L. fermentum* binding to mucin was twofold higher than that observed for the remaining *Lactobacillus* strains. When evaluated in an exclusion assay in the presence of genital pathogens, both *L. brevis* and *L. delbrueckii* ssp. *delbrueckii* significantly increased (twofold) *C. albicans* binding to mucin in comparison to the pathogen binding in the absence of lactobacilli (*p* < 0.05; **Figure 3A**). Similarly, all lactobacilli (excepting *L. plantarum*) enhanced *S. agalactiae* binding to mucin (three- to sixfold increase) (**Figure 3B**). No effects were observed for any of the tested lactobacilli on *N. gonorrhoeae* binding (*p* = 0.42; **Figure 3C**). Assessment of lactobacilli effects on pathogen binding in a displacement assay demonstrated that *C. albicans* binding to mucin is markedly inhibited (~80% inhibition; *p* < 0.001) by all tested *Lactobacillus* spp. (**Figure 4A**). None of the evaluated lactobacilli affected *S. agalactiae* and *N. gonorrhoeae* ability to bind to mucin (*p* = 0.51 and *p* = 0.36, respectively; **Figures 4B,C**).

### Co-aggregation of *Lactobacillus* spp. with Genital Pathogens

A co-aggregation assay was performed to evaluate whether *Lactobacillus* strains interact directly with genital pathogens. All lactobacilli co-aggregated with the tested genital pathogens, with distinct levels of interaction (**Table 2**). The highest co-aggregation score was observed for *L. fermentum*, which was able to interact with all pathogens, in especial *C. albicans* (score = 4). *L. paracasei* exhibited a high ability to co-aggregate with both *S. agalactiae* (score = 4) and *N. gonorrhoeae* (score = 3), but a low degree of co-aggregation with *C. albicans* (score = 1). Overall, *Lactobacillus* strains presented a low ability to interact with *S. agalactiae* (score = 1); excepting *L. fermentum* (score = 3) and *L. paracasei* (score = 4) to which it was observed a greater capacity to co-aggregate with this pathogen in comparison with the other tested lactobacilli.

**Table 2:** Co-aggregation scores for *Lactobacillus* spp. and genital pathogens.

*Lactobacillus* species	Aggregation score^∗^		
	*C. albicans*	*S. agalactiae*	*N. gonorrhoeae*
*L. acidophilus*	2	1	1
*L. brevis*	3	1	4
*L. delbrueckii* ssp. *delbrueckii*	1	1	1
*L. fermentum*	4	3	3
*L. paracasei*	1	4	3
*L. plantarum*	3	1	2
*L. rhamnosus*	3	1	1

*^∗^Mean co-aggregation scores of triplicate experiments. The microbial suspensions were evaluated and a score was attributed as follows: 0 = no aggregation, 1 = small aggregates comprising small visible clusters of bacteria, 2 = aggregates comprising larger numbers of bacteria, settling to the center of the well, 3 = macroscopically visible clumps comprising larger groups of bacteria which settle to the center of the well, 4 = maximum score allocated to describe a large, macroscopically visible clump in the center of the well. Aggregates were visualized under inverted light microscopy and results are expressed as aggregation score.*

### Antimicrobial Activity of *Lactobacillus* Strains against Genital Pathogens

We next assessed whether *Lactobacillus* spp. possess antimicrobial activity against genital pathogens. All lactobacilli inhibited pathogen growth, presenting inhibition zones ranging from 9.0 ± 0.8 to 19.6 ± 0.4 mm of diameter (**Table 3**; Supplementary Figures S1A–F). *L. rhamnosus* only reduced the growth of *C. albicans* in the area immediately above *Lactobacillus* growth, without producing a large inhibition zone (Supplementary Figure S1B). A similar activity was observed for *L. delbrueckii* ssp. *delbrueckii* when tested against *S. agalactiae* and *N. gonorrhoeae* (Supplementary Figures S1C,E).

**Table 3:** Growth inhibition zones of genital pathogens produced by *Lactobacillus* strains.

*Lactobacillus*	Inhibition zone (mm)^∗^		
	*C. albicans*	*S. agalactiae*	*N. gonorrhoeae*
*L. acidophilus*	15	16.3 ± 0.4	14.6 ± 0.4
*L. brevis*	13 ± 0.8	17.3 ± 0.4	19.3 ± 1.2
*L. delbrueckii* ssp. *delbrueckii^∗∗^*	–	–	–
*L. fermentum*	13 ± 0.8	14.3 ± 1.2	9 ± 0.8
*L. paracasei*	14	14.6 ± 0.4	13.6 ± 0.4
*L. plantarum*	14 ± 1.4	19.6 ± 0.4	17.3 ± 2.0
*L. rhamnosus^∗∗∗^*	–	15.3 ± 1.2	15

*^∗^Values represent the mean (mm) ± standard deviation (SD) of triplicate samples performed on three separate days. ^∗∗^Dashes (–) indicate that L. delbrueckii ssp. delbrueckii inhibited C. albicans, S. agalactiae, and N. gonorrhoeae growth only in the area above Lactobacillus growth without formation of an inhibition zone. ^∗∗∗^Dashes (–) indicate that L. rhamnosus inhibited C. albicans growth only in the area above Lactobacillus growth without formation of an inhibition zone.*

### Antimicrobial Activity of *L. fermentum* against Clinical Isolates of *Candida* spp.

As *L. fermentum* ATCC 23271 was the one that presented the greatest ability to form biofilm on surfaces, and the one that presented the greatest ability to co-aggregate with all tested pathogens, especially *C. albicans*, we carried out additional experiments in order to evaluate the antimicrobial effects of *L. fermentum* on clinical strains of *Candida* spp. Seven isolates of five species were tested in the same overlay inhibition assay and included *C. albicans* (two strains), *C. glabrata* (two strains), *C. parapsilosis* (one strain), *C. tropicalis* (one strain), and *C. krusei* (one strain). With the exception of *C. krusei*, all the remaining clinical strains had their growth inhibited by *L. fermentum*. A large inhibition zone was observed for one of the *C. albicans* isolates (18 mm), whereas the growth of the other *Candida* isolates were inhibited only in the area immediately above the *Lactobacillus* growth zone (**Table 4**).

**Table 4:** Antimicrobial activity of *L. fermentum* ATCC 23270 against clinical isolates of *Candida* spp.

*Candida* species (number of strains)	Strain designation	Inhibitory activity (inhibition zone in mm)^∗^
*C. albicans* (2)	VLAG	+ (18 ± 0.1)
	MRSM	+ (WZ)
*C. glabrata* (2)	FC 50	+ (WZ)
	CMLS	+ (WZ)
*C. parapsilosis* (1)	RCL	+ (WZ)
*C. tropicalis* (1)	RRL	+ (WZ)
*C. krusei* (1)	GSFO	–

*^∗^This value is the mean (mm) ± standard deviations of triplicate samples performed on three separate days; + denotes presence of inhibitory activity; WZ denotes that the growth was inhibited, but without zone formation; – denotes absence of inhibitory activity.*

## Discussion

Herein, we presented novel evidence on that *Lactobacillus* species that are less frequent in the female genital tract are potential probiotics. Although *L. fermentum* was not the best in terms of adhesion to HeLa cells and presented with similar effects to those of the other tested lactobacilli in regards of the exclusion and displacement assays, this bacteria was the one that presented the greatest ability to form biofilm and co-aggregate with all tested pathogens, especially *C. albicans*. We highlight that all lactobacilli tested only inhibited *C. albicans* in the displacement assay, thus suggesting a potential for treating vaginal infections caused by *C. albicans*.

Several parameters are considered in order to attribute probiotic properties to a microorganism. Criteria include (i) ability to adhere to host epithelial cells and mucus, (ii) biofilm production, (iii) capability to co-aggregate with bacterial pathogens, (iv) inhibition of pathogen binding to mucus and epithelial cells, and (v) antimicrobial activity (Andreu et al., 1995; Mastromarino et al., 2002; Martin et al., 2008; Van Tassell and Miller, 2011; Castro et al., 2013; Dhanani and Bagchi, 2013; Nishiyama et al., 2013).

We observed distinct adhesion profiles for the lactobacilli when tested with HeLa cells. This was expected as bacterial adhesion to cellular surfaces is multifactorial and involves different specific and non-specific interactions between bacterial and cell surface components. Non-specific mechanisms involve electrostatic and hydrophobic interactions, whereas the specific mechanisms are related to surface molecules, such as outer membrane proteins (Frece et al., 2005; Chen et al., 2007; Johnson-Henry et al., 2007; Zhang et al., 2013; Meng et al., 2014), exopolysaccharides (EPSs, Lebeer et al., 2011), lipoteichoic acids (Granato et al., 1999), peptidoglycans (Van Tassell and Miller, 2011), glycosaminoglycans (Martin et al., 2013), flagella, and fimbriae (Singh et al., 2013). This complex scenario results in species-specific adhesion (Deepika and Charalampopoulos, 2010; Messaoudi et al., 2012; Verdenelli et al., 2014) and may be related to the differences observed in the present study.

All the analyzed *Lactobacillus* species exhibited strong mucin binding at levels even higher (1 log_10_ difference) than those previously reported for other strains (Tallon et al., 2007). The main component of cervical mucus is mucin, which functions as a protective barrier against invasive pathogens in the uterus and vagina. An ideal probiotic may exhibit increased binding to mucus to facilitate its permanence and growth in the female genital tract, similar to their binding in the gastrointestinal tract (Perea Velez et al., 2007). Porcine intestinal mucin has been widely used to identifying the bind capacity of a certain bacteria to mucus (Tuomola et al., 1999; Izquierdo et al., 2008; Laparra and Sanz, 2009). We found that *L. acidophilus* ATCC 4356 and *L. paracasei* ATCC 335 poorly adhere to HeLa cells, although they are able to bind to mucin, suggesting these lactobacilli present mucin-specific surface lectins. On the other hand, *L. brevis* ATCC 367, *L. fermentum* ATCC 23271, *L. delbrueckii* ssp. *delbrueckii* ATCC 9645, *L. plantarum* ATCC 8014, and *L. rhamnosus* ATCC 9595 were able to adhere to HeLa cells and to bind to immobilized mucin, possibly due to their hydrophobic surfaces and/or expression of some adhesins. Mucin binding by probiotics is considered as a pre-requisite for creating a protective barrier against pathogen adhesion to epithelial cells by either occupying mucin-binding sites or producing antimicrobial compounds (Messaoudi et al., 2012; Zhang et al., 2013). It is possible that the greater the ability of *Lactobacillus* spp. to bind to mucin, the greater their ability to impair pathogen binding to the genital tract. It was previously described that microorganisms with a hydrophobic cell wall harbor a large variety of surface glycoproteins, increasing the probability of adhesion to cellular receptors (Kos et al., 2003) or to cell wall-anchored proteins (Polak-Berecka et al., 2014; Zhang et al., 2015). Indeed, adhesion-promoting proteins that are involved in the binding of distinct *Lactobacillus* species to mucin have been previously identified, including a mucin adhesion-promoting protein (MAPP) in *L. fermentum* strain 104R (Rojas et al., 2002), the elongation factor Tu in *L. johnsonii* strain La1 (Granato et al., 2004), and a fibronectin-binding protein (FbpB) in *L. acidophilus* strain NCFM (Hymes et al., 2016).

Interaction with mucin allows the lactobacilli to remain within the mucus layers, thereby contributing to the formation of multi-species biofilms (Wickstrom et al., 2013), which are essential for protection against intruding pathogens (Pi et al., 2011). Amongst the tested *Lactobacillus* species, *L. fermentum* ATCC 23271 was the only to produce moderate biofilm on plastic surface in comparison with other lactobacilli. Biofilm formation by *Lactobacillus* species is influenced by many variables, including the culture medium, the concentration of the inoculum, the tested strain, and even the chemical nature of the support used for the assay (Terraf et al., 2012). All tested genital pathogens adhered to mucin at the same level as the lactobacilli (>10^5^ CFU/ml). It is possible that in order to establish a genital infection, a successful pathogen has to overcome *Lactobacillus* colonization thus promoting host cell adherence and/or invasion. To test this hypothesis, we performed two competitive binding assays to mucin, exclusion (*Lactobacillus* added and then, pathogen) and displacement (pathogen added and then, *Lactobacillus*). Interestingly, the exclusion assay indicated lactobacilli enhance pathogen adherence to mucin. A previous study reported that some lactobacilli isolated from the vaginal tract enhance the adherence of *C. albicans* and *Actinomyces neuii* to human epithelial cells *in vitro* (Martin et al., 2012). The same study suggested that secretion of aggregation proteins by lactobacilli might favor a positive interaction of pathogens within host cells. Additional studies have suggested that expression of EPSs may enhance the interaction of pathogens with host cells (Marcotte et al., 2004; Ruas-Madiedo et al., 2006; Macias-Rodriguez et al., 2009). Apparently, this increased adhesion of pathogens might be explained by the increased binding of pathogens to specific EPSs, which in turn, enhanced adherence to mucus (Ruas-Madiedo et al., 2006). Although these events have been observed for enteropathogens, they may also occur for genital pathogens.

On the other hand, all lactobacilli inhibited the adhesion of *C. albicans* in the displacement assay, suggesting they could potentially be used to complement the treatment of infections caused by this pathogen, at least as adjuvants. *C. albicans* displacement may be attributed to the production of antimicrobial substances by the lactobacilli, as supported by the antimicrobial activity assay. Furthermore, *Lactobacillus* species exhibited inhibitory effects against yeasts at low concentrations (10^5^ CFU/ml). This finding may be of clinical relevance for probiotic therapy in immunocompromised patients in whom high concentrations of these microorganisms can trigger clinical complications (Kirjavainen et al., 1999; Apostolou et al., 2001; Ren et al., 2012). None of the tested lactobacilli affected either *S. agalactiae* or *N. gonorrhoeae* in the displacement assay. It is possible that these bacteria express proteins (or other components) that bind with high affinity to mucin, and consequently, this has hindered the displacement effect for the lactobacilli analyzed. In fact, adhesins involved in host cell colonization and in the interaction to host extracellular components have been described for both pathogens (Edwards and Apicella, 2004; Lalioui et al., 2005).

In the present study, lactobacilli presented with different co-aggregation activities. *L. fermentum* co-aggregated most strongly with the genital pathogens *C. albicans*, *N. gonorrhoeae*, and *S. agalactiae*. In agreement with previous studies, *Lactobacillus* species, including *L. acidophilus*, *L. gasseri*, amongst others, exhibit different co-aggregation activities with the same pathogens, suggesting a species-specific process (Boris et al., 1998; Osset et al., 2001). The formation of co-aggregates has been suggested as a key event for the elimination of pathogens as this close interaction allows the lactobacilli to create an adverse microenvironment for pathogens by delivering antimicrobial substances in a localized manner, and thus, impairing epithelial colonization (Mastromarino et al., 2002; Younes et al., 2012). Herein, all tested *Lactobacillus* strains were inhibitory against the tested genital pathogens. This is in agreement with previous reports showing that *Lactobacillus* species, including those assessed in our study, are able to produce antibacterial metabolites, such as bacteriocins (Todorov et al., 2011), biosurfactants, organic acids, and H_2_O_2_ (Verdenelli et al., 2014); which can act in synergy, reducing pathogen survival.

The probiotic properties of *L. fermentum* ATCC 23271 observed for the reference strain of *C. albicans* as well as the growth inhibition of *Candida* clinical isolates suggest that it is possible this *Lactobacillus* may be useful for the treatment or prophylaxis of vaginal candidiasis. Interestingly, *L. fermentum* inhibited the growth of two of the most relevant *Candida* species involved in vaginal candidiasis: *C. albicans*, which can be found in 80–90% of cases and *C. glabrata*, which is the second most common species, being responsible for 5–15% of the vaginal candidiasis cases (Ferrer, 2000; Sobel, 2007). Indeed, *L. fermentum* has been previously shown to inhibit pathogen growth. In a recent study, the growth and virulence of *Escherichia coli* and *Gardnerella vaginalis* were inhibited by the *L. fermentum* strain SK5 (Kaewnopparat et al., 2013). Similar effects were observed for the *L. fermentum* strain Ess-1, which was shown to inhibit *C. albicans* and *C. glabrata* growth (Rönnqvist et al., 2007) and for *L. fermentum* HV6b MTCC 10770, which was able to reduce the growth of *Bacteroides*, *G. vaginalis*, *Mobiluncus*, *Staphylococcus* spp., and *Streptococcus* spp *L. fermentum* antimicrobial actions have been attributed to its ability to produce a bacteriocin-like compound (Sabia et al., 2014) and to secrete the antimicrobial peptide fermenticin HV6b (Kaur et al., 2013). Additionally, Pascual et al. (2010) demonstrated that *L. fermentum* L23 presents *in vivo* probiotic actions against vaginitis by treating and preventing *E. coli*-induced infections.

Collectively, our data show for the first time that *L. fermentum* ATCC 23271 is a prominent probiotic, exhibiting the highest levels of adhesion to HeLa cells and binding to mucin, in addition to antimicrobial properties against pathogens of importance to female genital tract infections, particularly against *Candida* spp. Herein, *L. fermentum* presented with the best probiotic profile in comparison with the other tested lactobacilli species. Finally, we suggest that *L. fermentum* could be used alone or in combination with other species in order to treat genital infections.

## Author Contributions

MC designed and performed the experiments, contributed to data interpretation, manuscript writing, and approval of the final version for publication; FN, MA, EdS, and MB performed the experiments, contributed to data interpretation, and approval of the final version for publications; AM and TF contributed to data interpretation and revised the manuscript; EF and JG contributed to data interpretation, revised the manuscript, and approved the final version for publication; VM-N conceived the study, participated in its design and coordination, and critically revised the manuscript.

## Conflict of Interest Statement

The authors declare that the research was conducted in the absence of any commercial or financial relationships that could be construed as a potential conflict of interest.
