# Supplementary material for: Lactobacillus fermentum ATCC 23271 Displays In vitro Inhibitory Activities against Candida spp
Source: Front Microbiol. 2016 Oct 27;7:1722. doi: 10.3389/fmicb.2016.01722 (PMC5082230; doi:10.3389/fmicb.2016.01722)
Supplement: Supplementary file 1 [file Image_1.PDF]

Supplementary Material

Lactobacillus fermentum ATCC 23271 Displays *in vitro* Probiotic Properties Against *Candida* spp.

Monique S. do Carmo[1], Francisca Maria F. Noronha[2], Mariana O. Arruda[1], Ennio Patrezzi da S. Costa[2], Maria Rosa Q. Bomfim[2], Andrea S. Monteiro[2], Thiago A. F. Ferro[2], Elizabeth S. Fernandes[2,3], Jorge A. Girón[4], Valério Monteiro-Neto[1,2,*]

[1]Centro de Ciências Biológicas e da Saúde, Universidade Federal do Maranhão, São Luís, MA, Brazil

[2]Laboratório de Biologia Molecular de Microrganismos, Universidade CEUMA, Centro de Ciências da Saúde, São Luís, MA, Brazil

[3]Vascular Biology and Inflammation Section, Cardiovascular Division, King's College London, London, United Kingdom

[4]Centro de Detección Biomolecular, Benemérita Universidad Autónoma de Puebla, Ciudad Universitaria, Puebla, PUE, México.

*** Correspondence:**

Valério Monteiro-Neto

valerio.monteiro@ceuma.br

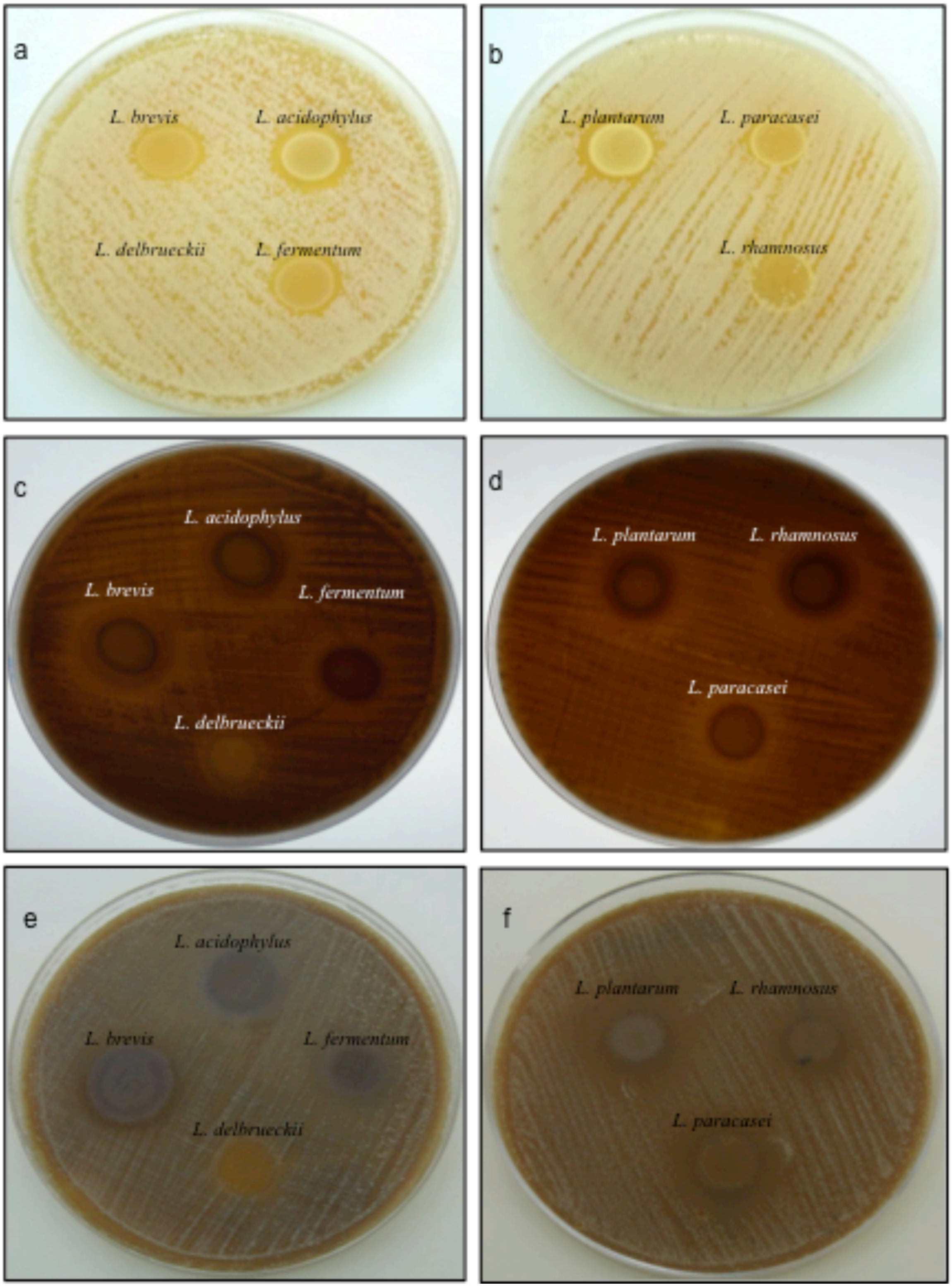

Supplementary Figure 1. Inhibition growth produced by *Lactobacillus* against genital pathogens. (a and b) *C. albicans*; (c and d) *S. agalactiae*; (e and f) *N. gonorrhoeae*. The assay was performed in triplicate.
